# Prenatal Alcohol Exposure as a Potential Contributor to Hydranencephaly: Clinical and Neurodevelopmental Perspectives

**DOI:** 10.1002/ccr3.73309

**Published:** 2026-08-09

**Authors:** Chukwuka Elendu, Botiwuoluwa M. Ogunmola, Adetokunbo A. Adesina, Alexandra U. Akudolu, Favour K. Mbakwe

**Affiliations:** ^1^ Federal University Teaching Hospital Owerri Nigeria; ^2^ Indiana University Bloomington Bloomington Indiana USA; ^3^ Buckinghamshire NHS Trust Aylesbury UK; ^4^ Queen Alexandra Hospital Portsmouth UK; ^5^ Obafemi Awolowo University Teaching Hospitals Complex Ile‐Ife Nigeria

**Keywords:** cerebrovascular disruption, fetal alcohol spectrum disorders, hydranencephaly, neurodevelopmental injury, prenatal alcohol exposure

## Abstract

Hydranencephaly is traditionally attributed to prenatal vascular disruption; however, chronic prenatal alcohol exposure may contribute through neurotoxicity, placental dysfunction, oxidative stress, and vascular injury. Given the biological plausibility of this association, alcohol abstinence during pregnancy remains essential, and further studies are required to clarify its role in hydranencephaly.

Dear Editor,

We read with great interest the recent report describing a neonate with hydranencephaly born to a mother with chronic alcohol consumption throughout gestation [1].

Hydranencephaly is a devastating encephaloclastic disorder characterized by near‐complete absence of the cerebral hemispheres, which are replaced by cerebrospinal fluid while portions of the brainstem, thalami, basal ganglia, and cerebellum are preserved [2]. The condition is most commonly attributed to bilateral disruption of fetal internal carotid artery circulation during a critical stage of neurodevelopment, resulting in extensive ischemic destruction of cerebral tissue [3, 4]. This vascular hypothesis is supported by experimental studies demonstrating hydranencephalic changes following fetal carotid artery occlusion [5]. However, hydranencephaly is increasingly recognized as a heterogeneous disorder with infectious, toxic, genetic, and environmental factors also implicated in its pathogenesis [3, 6].

Within this context, prenatal alcohol exposure warrants careful consideration because alcohol readily crosses the placenta, and the fetus has limited capacity for ethanol metabolism, resulting in prolonged exposure of developing neural tissues to ethanol and its toxic metabolites [7]. This exposure disrupts key neurodevelopmental processes, including neuronal proliferation, migration, differentiation, synaptogenesis, and programmed cell survival through mechanisms involving neurotransmitter dysregulation, oxidative stress, mitochondrial dysfunction, altered growth factor signaling, and epigenetic modification [7, 8, 9]. Collectively, these effects contribute to the spectrum of structural and functional abnormalities recognized as fetal alcohol spectrum disorders (FASD), ranging from subtle neurocognitive deficits to major congenital malformations [10].

Although hydranencephaly is not traditionally considered a manifestation of FASD, emerging evidence suggests plausible biological links between prenatal alcohol exposure and severe cerebral injury (Figure 1). Ethanol exposure during critical periods of fetal brain development can induce widespread neurodegeneration through disruption of N‐methyl‐D‐aspartate and γ‐aminobutyric acid receptor signaling, oxidative stress, mitochondrial dysfunction, and inflammatory pathways, resulting in substantial neuronal loss [8, 11, 12]. Moreover, alcohol‐related endothelial dysfunction, impaired angiogenesis, altered placental perfusion, and dysregulation of vascular growth factors may increase susceptibility to ischemic cerebral injury, providing a potential mechanistic bridge between alcohol exposure and the vascular pathology implicated in hydranencephaly [13, 14].

**FIGURE 1:**
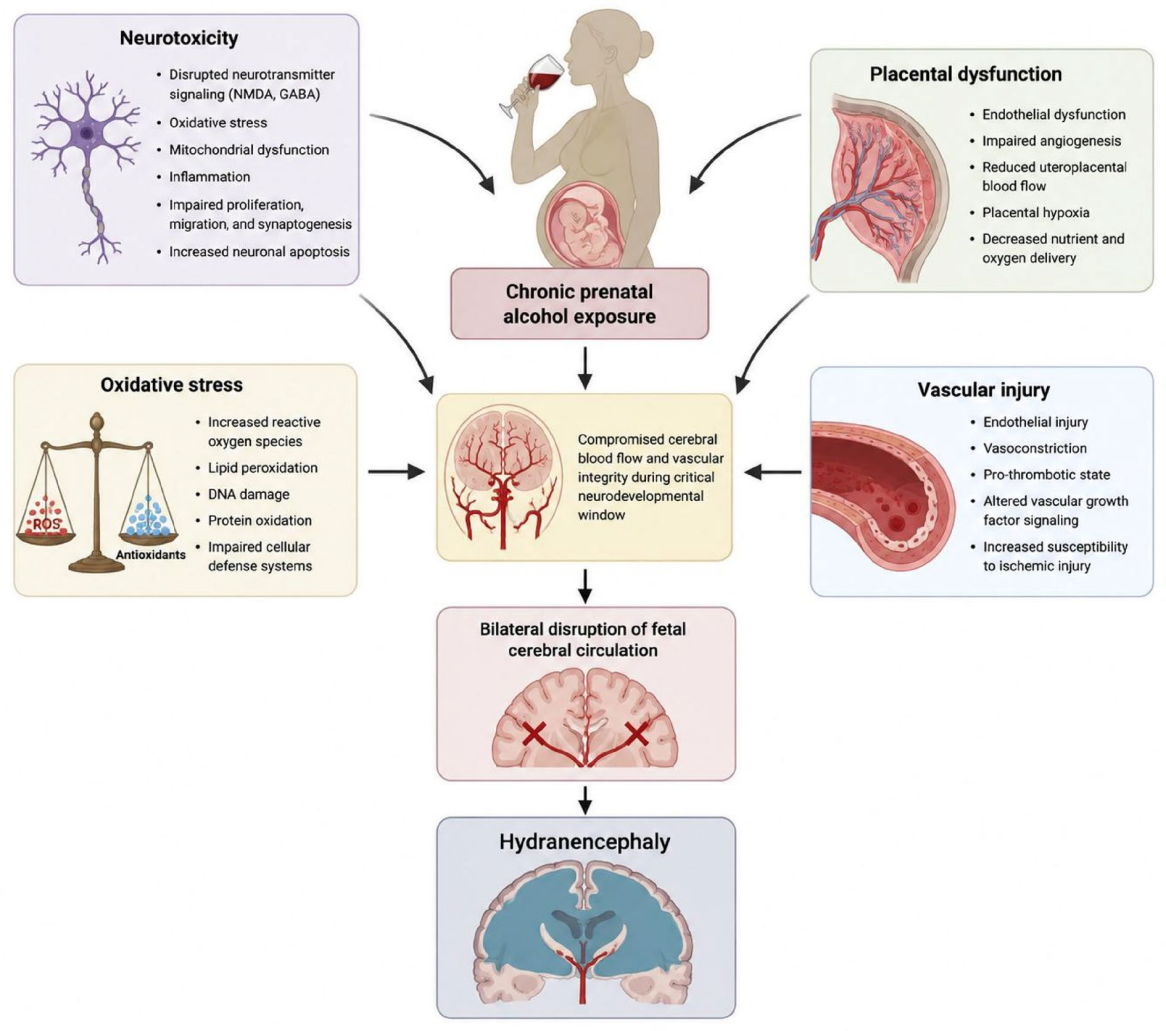
Proposed mechanisms through which chronic prenatal alcohol exposure may contribute to the pathogenesis of hydranencephaly via neurotoxicity, oxidative stress, placental dysfunction, and vascular injury.

The potential contribution of prenatal alcohol exposure to hydranencephaly through vascular mechanisms warrants further consideration. Maternal ethanol exposure has been associated with placental vascular abnormalities, impaired uteroplacental blood flow, placental hypoxia, and reduced nutrient delivery, all of which may compromise fetal oxygenation during critical stages of cerebral development [14, 15]. In addition, alcohol‐induced vasoconstriction and endothelial injury have been implicated in fetal vascular disruption syndromes, providing further biological plausibility for a role in severe encephaloclastic lesions [16]. Although causality cannot be inferred from a single case, the coexistence of chronic maternal alcohol consumption and hydranencephaly highlights the need for further investigation into whether alcohol contributes to a subset of otherwise unexplained cases.

Hydranencephaly is generally thought to result from destructive events occurring after formation of the cerebral hemispheres, most commonly during the second trimester [3, 4]. The documented maternal alcohol exposure spanned this critical period of rapid brain growth, neuronal migration, and vascular maturation, potentially amplifying the impact of ischemic insults. Accordingly, alcohol may be viewed not only as a direct neurotoxin but also as a factor that increases susceptibility to catastrophic cerebral injury through interactions involving oxidative stress, vascular instability, placental dysfunction, and impaired neuroprotection [9, 13].

Establishing causation in hydranencephaly remains challenging given its association with diverse etiologies, including congenital infections, twin gestations, thromboembolic events, maternal toxic exposures, and rare genetic disorders [3, 6]. Although negative infectious screening increases the relevance of the documented alcohol exposure, the absence of advanced genetic testing and detailed fetal neuroimaging precludes definitive etiological attribution. Moreover, increasing recognition of monogenic disorders affecting cerebrovascular development underscores the value of comprehensive genomic evaluation in apparently sporadic cases [17]. Future reports would benefit from incorporating chromosomal microarray analysis, exome sequencing, placental examination, and magnetic resonance imaging to facilitate more robust etiological characterization.

From a clinical perspective, the detection of fetal hydrocephalus during late gestation highlights the diagnostic challenge posed by hydranencephaly, which may mimic severe hydrocephalus following extensive cerebral tissue loss (Figure 2A,B) [18]. Early anomaly scans, serial fetal neurosonography, and, when indicated, fetal magnetic resonance imaging can improve diagnostic accuracy and facilitate differentiation from porencephaly, alobar holoprosencephaly, and other cystic cerebral abnormalities [18, 19]. Timely diagnosis is essential for informed parental counseling, delivery planning, neonatal management, and prognostic assessment.

**FIGURE 2:**
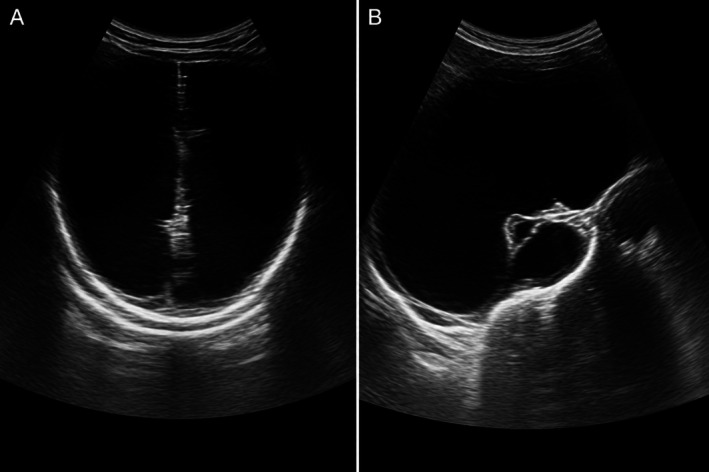
Cranial ultrasonographic views of hydranencephaly: (A) near‐complete replacement of the cerebral hemispheres by fluid‐filled spaces; (B) extensive loss of cerebral parenchyma with preservation of deep midline structures. This image is intended for educational and illustrative purposes only and does not represent the original patient imaging. The radiologic patterns were adapted from the clinical descriptions reported in the referenced case report [1].

Despite longstanding recommendations advocating complete abstinence, many women continue to consume alcohol before pregnancy recognition or throughout gestation, making prenatal alcohol exposure a persistent and preventable cause of fetal morbidity [20]. Consequently, the burden of alcohol‐related fetal injury is likely underestimated, particularly in settings with limited screening and surveillance systems. Severe neurological outcomes such as hydranencephaly reinforce the importance of prevention and support current recommendations that no safe level of alcohol exposure during pregnancy has been established [10, 20].

Systematic documentation of maternal exposure histories and aggregation of similar cases through multicenter registries may help determine whether prenatal alcohol exposure is associated with hydranencephaly beyond chance alone. Such questions will require well‐designed epidemiological studies that account for potential confounders, including socioeconomic factors, nutritional status, concurrent substance use, maternal comorbidities, and genetic susceptibility. Until stronger evidence emerges, causal inferences should remain cautious while acknowledging the biological plausibility of a contributory role.

In summary, bilateral vascular disruption remains the most widely accepted mechanism underlying hydranencephaly; however, current evidence suggests several biologically plausible pathways through which chronic prenatal alcohol exposure may contribute to its pathogenesis, including neurotoxicity, placental dysfunction, oxidative stress, and vascular injury. Despite longstanding recommendations advocating complete abstinence, many women continue to consume alcohol before pregnancy recognition or throughout gestation, making prenatal alcohol exposure a persistent and preventable cause of fetal morbidity.

## Author Contributions

**Botiwuoluwa M. Ogunmola:** investigation, validation, writing – original draft. **Chukwuka Elendu:** conceptualization, investigation, data curation, methodology, formal analysis, writing – original draft, writing – review and editing, visualization, supervision, validation, project administration. **Alexandra U. Akudolu:** investigation, validation, writing – review and editing. **Adetokunbo A. Adesina:** formal analysis, validation, writing – review and editing. **Favour K. Mbakwe:** supervision, validation, writing – review and editing.

## Funding

The authors have nothing to report.

## Disclosure

The views expressed in this report are solely those of the authors and do not represent the official positions of any affiliated institutions.

## Ethics Statement

Ethical approval was not required, as this letter does not involve new patient data, human subject research, or original clinical investigation.

## Consent

Written informed consent for publication was obtained where applicable. This letter does not include new patient data.

## Conflicts of Interest

The authors declare no conflicts of interest.

## Data Availability

No datasets were generated or analyzed for this letter.
